# Microhabitat Use of Temminck’s Tragopan (*Tragopan temminckii*) During the Breeding Season in Laojunshan National Nature Reserve, Western China

**DOI:** 10.3390/biology15030221

**Published:** 2026-01-25

**Authors:** Li Zhao, Ping Ye, Benping Chen, Lingsen Cao, Yingjian Tian, Yiming Wu, Yiqiang Fu, Wenbo Liao

**Affiliations:** 1 Key Laboratory of Southwest China Wildlife Resources Conservation (Ministry of Education), China West Normal University, Nanchong 637009, China; lizhao_688@163.com (L.Z.); skyeping@cwnu.edu.cn (P.Y.); ls_11242024@163.com (L.C.); yingjian@126.com (Y.T.); yimingwu@126.com (Y.W.); 2 Key Laboratory of Artificial Propagation and Utilization in Anurans of Nanchong City, China West Normal University, Nanchong 637009, China; 3 Laojunshan National Natural Reserve, Pingshan 645350, China; chenbenping2026@163.com; 4 Key Laboratory of Ecological Adaptation in Amphibian in Sichuan Province, China West Normal University, Nanchong 637009, China

**Keywords:** habitat use, Temminck’s tragopan, Laojunshan Nature Reserve, breeding season

## Abstract

**Simple Summary:**

The Temminck’s Tragopan (*Tragopan temminckii*), a protected pheasant species inhabiting mountainous forests in China, requires specific habitat features during the breeding season. We examined its microhabitat preferences in the Laojunshan National Nature Reserve, Sichuan Province. Our results indicate that the species strongly prefers areas characterized by tall trees and bamboo, dense overhead cover, thick leaf litter, and tall shrubs, while avoiding habitats dominated by dense, tall grasses. Principal Component Analysis revealed that bamboo structure, tree structure, and proximity to water sources and forest edges are key environmental factors influencing habitat selection. Effective conservation of this species therefore requires forest management practices that maintain this specific combination of habitat characteristics to support its reproduction and survival.

**Abstract:**

Habitat utilization is a critical determinant of animal survival and reproductive success. Clarifying species-specific habitat preferences provides essential insights into ecological requirements and forms the basis for sound conservation planning. The Temminck’s Tragopan (*Tragopan temminckii*), a medium-sized, sexually dimorphic pheasant endemic to montane forests of central and southern China, is classified as a nationally protected Class II species. Nevertheless, its fine-scale habitat selection during the breeding season remains inadequately documented. In 2024, we conducted a field investigation in the Laojunshan National Nature Reserve, Sichuan Province, to examine microhabitat use during this critical period. Our analysis revealed a significant preference for sites characterized by greater tree and bamboo height, higher canopy and bamboo cover, increased litter coverage, and taller shrub layers. In contrast, the species consistently avoided locations dominated by dense, tall herbaceous vegetation. Principal Component Analysis identified six principal components, collectively explaining 71.78% of the total environmental variance. The first component was primarily associated with bamboo structural attributes, the second with tree-layer structure, and the third with proximity to forest edges and streams. These findings indicate that effective conservation of this pheasant requires targeted forest management practices that preserve this specific suite of habitat characteristics, which are essential for ensuring reproductive success and long-term population viability.

## 1. Introduction

The escalating global human population and rapid socioeconomic development have resulted in increasingly severe degradation of natural resources [1,2,3]. Global biodiversity is declining due to intensifying anthropogenic disturbances [4,5,6,7,8]. Protecting wildlife habitats is widely regarded as the most effective conservation strategy, as habitat loss and fragmentation accelerate species extinctions by reducing the availability of essential resources and suitable conditions for survival and reproduction [9,10,11,12,13]. However, the lack of fundamental ecological data for many endangered species hinders the development of appropriate conservation strategies. Consequently, research on habitat use by ground-dwelling birds is of particular importance [14,15].

Habitat use is a key adaptive strategy that enables animals to cope with environmental heterogeneity, ultimately influencing individual fitness, population distribution, and community stability [16,17,18,19]. Habitat can be broadly categorized across different spatial scales, including macrohabitats (e.g., geographic ranges) and microhabitats (e.g., specific sites selected within home ranges) [16]. Microhabitat characteristics directly shape fine-scale habitat preferences, encompassing features such as topography, understory vegetation density, and proximity to water sources [20]. However, most existing studies have focused on macro-scale patterns or examined habitat use during a single season, with limited attention to microhabitat selection across different life-history stages or seasons [21,22]. In reality, microhabitat use is governed by a complex interplay of resource availability, predation risk, and behavioral trade-offs [23,24]. For instance, individuals may prioritize concealment and access to critical resources to enhance reproductive success during the breeding season [25], whereas during the non-breeding season, the emphasis may shift toward maximizing energy intake and minimizing predation risk [26]. Therefore, examining habitat selection at the microhabitat scale across different life-history stages is essential for understanding species’ ecological adaptations and for informing effective habitat management strategies [27].

The Temminck’s Tragopan (*Tragopan temminckii*), a medium-sized, sexually dimorphic pheasant endemic to the montane forests of central and southern China, is listed as a nationally protected species and classified as Near Threatened on the IUCN Red List [28]. It primarily inhabits dense, moist temperate forests with a well-developed understory of bamboo and rhododendron, typically at elevations of 1800–3900 m [29]. The species exhibits striking sexual dimorphism: males display vibrant orange-red plumage with numerous white-spotted blue facial skin wattles and fleshy horns during courtship, whereas females are predominantly brown with cryptic barring that provides effective camouflage during nesting. The Temminck’s Tragopan is omnivorous, feeding mainly on herbs and ferns in spring and winter, and on mature fruits in summer and autumn [30,31]. Previous studies on this species have primarily focused on behavioral and ecological aspects, including activity rhythms [32,33], niche characteristics [34], flocking behavior [35], roosting and foraging habitats [36,37], and breeding ecology [38]. However, systematic analyses of microhabitat use remain scarce. This knowledge gap limits a comprehensive understanding of the species’ ecological requirements and constrains effective conservation planning. To address this gap, we conducted a systematic field investigation of Temminck’s Tragopan microhabitat use during the breeding season (April–June) in Laojunshan National Nature Reserve, Sichuan Province. Specifically, our objectives were to (1) identify key environmental variables influencing microhabitat use, and (2) examine habitat preferences and their potential driving factors. Our findings provide a scientific basis for refined conservation management and offer theoretical insights into the conservation of microhabitats for ground-dwelling birds.

## 2. Materials and Methods

### 2.1. Study Area

This research was conducted in the Laojunshan National Nature Reserve, located in Pingshan County, Sichuan Province, China, from April to July 2024. The study area covers approximately 35 km^2^ within the Xiaoliang Mountains (28°45′ N, 103°56′ E) and spans an elevational gradient of 1000–2010 m. The region experiences a mean annual temperature of 12.5 °C and receives an average annual precipitation of approximately 1500 mm. Vegetation in the reserve is dominated by evergreen broad-leaved forest, with tree species including *Castanopsis delavayi*, *C. omeiensis*, *Davidia involucrata*, and *Cyclobalanopsis myrsinifolia*. The shrub layer is well developed and includes *Eurya loquiana, Alangium chinense, Rhododendron hunnewellianum,* and *Camellia oleifera*, while the understory is characterized by dense bamboo thickets, primarily *Chimonobambusa quadrangularis.*

### 2.2. Fieldwork

**Figure 1:**
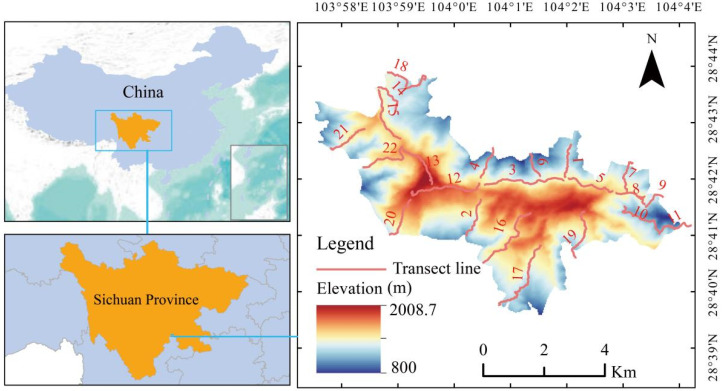
Location of the study area and distribution of transects for surveying Temminck’s Tragopan (*Tragopan temminckii*) microhabitat use in Laojunshan Nature Reserve, Sichuan Province, China.

We employed a systematic transect-based approach to investigate the foraging or mating habitats of *T. temminckii*. A total of 22 permanent transects, ranging from 1 to 3 km in length, were strategically distributed throughout the reserve to capture representative topographic variation, including ridge, slope, and valley positions, as well as structural diversity across vegetation types. These transects were surveyed twice during the breeding season (surveys separated by more than a month) to ensure adequate temporal coverage of roosting site use (Figure 1). During daylight hours, all visual or auditory detections of tragopan individuals, as well as fresh indirect signs such as feathers, droppings, or foraging traces observed within 100 m on either side of each transect, were meticulously recorded and georeferenced. Auditory detections were followed by careful approach to locate the individual. Once the individual was visually located, the spot was centered on its actual position. At each detection point, a 10 m × 10 m vegetation plot was established and centered on the location of occurrence. Within each quadrat, we measured a suite of habitat variables, including topographic variables (elevation, slope, and aspect), vegetation variables (tree height, tree diameter, tree cover, shrub height, shrub cover, herb height, herb cover, and litter cover), and distance variables (distance to the nearest stream, road, and forest edge). The “roads” were defined as any path accessible to motor vehicles. “Forest edge” was defined as the transition zone between forest canopy closure and open habitat. To characterize fine-scale habitat structure, four 1 m × 1 m subquadrats were placed at the corners of the main plot to measure bamboo height and bamboo cover. For comparison of habitat availability, an additional control plot was established 100 m away from each utilization plot in a randomly selected direction within the same territory, where identical environmental variables were recorded. To minimize topographic confounding effects, control plots were randomly positioned within the same elevation band as their corresponding utilization plots. In conclusion, all of them the general survey protocol, including the measurement methods for each habitat variable, followed that described in [39].

### 2.3. Statistical Analyses

Data normality and homogeneity of variance were assessed using the Shapiro–Wilk test and Levene’s test, respectively. As most variables deviated significantly from parametric assumptions, differences in foraging or mating habitat characteristics between used sites and randomly selected control sites were analyzed using the non-parametric Mann–Whitney U test. Chi-square tests were applied to evaluate potential selectivity in roosting tree species and to assess non-random orientation patterns of roosting branches. To identify the primary environmental drivers of habitat selection, we conducted a Principal Component Analysis (PCA) on 16 standardized habitat variables. Principal components with eigenvalues greater than 1, based on the Kaiser–Guttman criterion, were retained for ecological interpretation. These retained components were subsequently used as predictors in binomial logistic regression models to further quantify their influence on roosting habitat selection.

Model selection was guided by the Akaike Information Criterion corrected for small sample sizes (AICc), whereby all possible combinations of the retained principal components were generated and evaluated. Candidate models with ΔAICc < 2 relative to the top-ranked model were incorporated into a multimodel inference framework implemented using the MuMIn package in R version 4.3.0 [40], allowing the calculation of model-averaged parameter estimates with improved robustness and reduced sampling bias. The discriminative performance of the final averaged model was assessed by calculating the Area Under the Receiver Operating Characteristic Curve (AUC). All statistical analyses were conducted in R version 4.3.0, with statistical significance set a priori at α = 0.05, and continuous variables summarized as mean ± standard error.

## 3. Results

**Figure 2:**
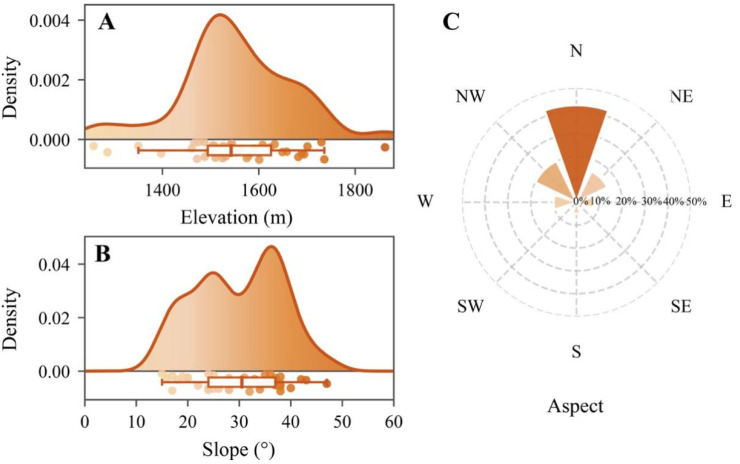
Topographic variation in microhabitat use by the Temminck’s tragopan during the breeding season (n = 40). (**A**) Kernel density plot of the used elevations. (**B**) Kernel density plot of the used slopes. (**C**) Radar chart showing the proportion of locations across aspects. Aspect categories: N (north), NE (northeast), E (east), SE (southeast), S (south), SW (southwest), W (west), and NW (northwest).

Habitat use by the Temminck’s Tragopan was examined from April to July 2024. All recorded territories and their associated habitats were confined to evergreen broad-leaved forest within the study area and were not observed in coniferous plantations, farmland, or settlements. These sites spanned nearly the entire elevational range of the reserve, from 1256 to 1862 m above sea level. As tragopans were not detected outside broad-leaved forest, only random plots within this habitat type were used for comparison with sites utilized by the birds. Compared with randomly selected control plots, the Tragopan showed a significant preference for habitats with slopes of approximately 30° (Figure 2A,B) and closer proximity to streams (Table 1). Aspect analysis revealed an overall preference for shady and semi-shady aspects (Figure 2C). In addition, *T. temminckii* significantly preferred habitats characterized by greater tree and bamboo height (all *p* < 0.001), higher tree and bamboo cover (all *p* < 0.001), increased litter cover (*p* < 0.001), and taller shrubs (*p* = 0.004). In contrast, the species avoided areas with dense and tall herbaceous vegetation, as indicated by lower herb cover and height at used sites (Table 1).

To further examine habitat use by the Temminck’s Tragopan, a Principal Component Analysis (PCA) was conducted on 15 microhabitat variables, with components having eigenvalues greater than 1 retained for subsequent analyses. These retained components were then incorporated into binomial logistic regression models, followed by model averaging of those with ΔAICc < 2. The combined results confirmed significant differences in microhabitat utilization between used and available sites. During the breeding season, six principal components were retained, collectively explaining 71.78% of the total environmental variance (Table 2). The first principal component (RC1) accounted for 22.72% of the variance and primarily reflected bamboo structural characteristics (loadings: bamboo height = 0.892; bamboo cover = 0.909). The second component (RC2) explained 15.07% of the variance and represented tree-layer structure (loadings: tree height = 0.874; tree cover = 0.844). The third component (RC3) accounted for 12.64% of the variance and was associated with proximity to streams and forest edges (loadings: distance to stream = 0.895; distance to forest edge = 0.860). The remaining components (RC4, RC5, and RC6) were mainly related to elevation and distance to roads, shrub cover, and slope, respectively.

**Figure 3:**
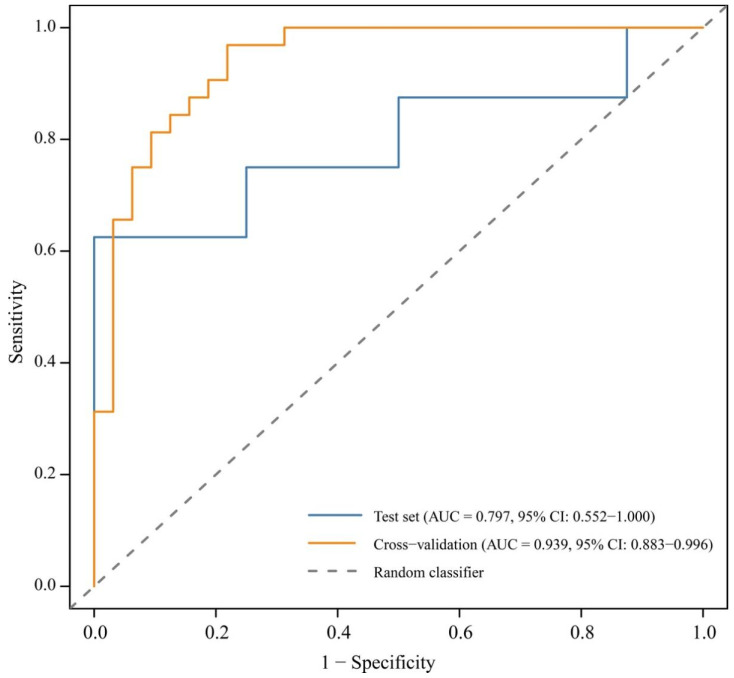
ROC curves assessing model performance for habitat use by the Temminck’s tragopan during breeding seasons. (Note: ROC represents receiver operating characteristic; AUC represents the area under the ROC curve).

A logistic regression model incorporating PCA scores was constructed to predict microhabitat use (Table 3). In the best-supported model (ΔAICc < 2), PC1 (bamboo structure) and PC2 (tree structure) exerted highly significant positive effects on habitat utilization (PC1: β = 4.023, *p* = 0.001; PC2: β = 3.321, *p* = 0.002). PC3 (distance to streams and forest edges) exhibited a marginally negative effect (β = −2.039, *p* = 0.075), whereas PC4 and PC6 were not statistically significant (*p* > 0.05). Model performance assessed using Receiver Operating Characteristic (ROC) curves yielded AUC values of 0.797 for the test dataset and 0.939 for cross-validation, indicating strong predictive accuracy and good generalization performance (Figure 3).

## 4. Discussion

Our study demonstrates that during the breeding season, *T. temminckii* exhibits a pronounced preference for microhabitats characterized by moderately steep slopes, well-developed overstory tree and bamboo layers, higher litter cover, sparse herbaceous vegetation, and close proximity to perennial streams. Among these variables, the structural complexity of the bamboo and tree layers emerged as the most influential drivers of habitat selection, a pattern consistent with other sympatric pheasants such as the *Arborophila rufipectus* [41] and *Lophura nycthemera* (unpublished data). In the following section, we interpret these findings in the context of the foraging, nesting, and anti-predator strategies typical of understory-dependent pheasants, and discuss their implications for breeding ecology and conservation management.

The microhabitat use strategy of *T. temminckii* in subtropical montane ecosystems reflects a dual response to environmental heterogeneity, integrating both stable and flexible components. Our findings reveal a pronounced and consistent preference for specific topographic features, including elevations of 1256–1862 m, slopes averaging approximately 30°, and predominantly shady to semi-shady aspects. This pattern is consistent with a broader ecological strategy observed in many phasianids, which often select geomorphologically stable sites to minimize predation risk and reduce exposure to anthropogenic disturbance [42,43,44,45]. In addition, *T. temminckii* exhibited a clear preference for habitats in close proximity to perennial streams, a behavior likely associated with ensuring reliable access to water for both adults and chicks during the breeding season. Similar dependencies on water availability have been documented in other galliform species [46]. The significant negative association with the principal component representing distance to water sources (PC3) further underscores the critical role of hydrological proximity in shaping habitat use by this species.

In contrast to its relatively stable topographic template, *T. temminckii* exhibits pronounced and specific preferences for vegetation structure during the breeding season [47]. The species shows strong selection for habitats with well-developed vertical stratification, characterized by tall bamboo and trees with extensive cover (PC1 and PC2), while actively avoiding areas dominated by dense and tall herbaceous layers. These selected structural attributes are functionally integral to reproductive success. Dense bamboo thickets and a tall tree canopy provide critical concealment, reducing detection and access by predators [44,48]. Concurrently, deep leaf litter supports a higher abundance of invertebrate prey, supplying essential protein for breeding adults and developing chicks [49]. Moreover, the avoidance of dense herbaceous cover likely serves a dual function: it enhances the visual field for early predator detection by attending adults and promotes a drier microclimate at nest sites. Reduced ambient humidity may, in turn, lower the risk of fungal pathogen proliferation and the physical degradation of nest materials [50,51]. Collectively, this seasonally specialized vegetation profile appears to optimize concealment, nutritional availability, and nest-site hygiene during the critical breeding period.

The findings of this study underscore a critical ecological linkage between fine scale habitat selection by *T. temminckii* and its demographic resilience, illustrating a pronounced case of life stage dependent adaptation to environmental heterogeneity. Our analysis indicates that the species long term persistence is fundamentally contingent upon access to structurally complex and spatially heterogeneous understory environments, with distinct shifts in resource requirements occurring across key life history stages, most notably during the breeding period [52]. However, this adaptive reliance is increasingly threatened across the species range by pervasive habitat loss, fragmentation, and other anthropogenic disturbances [53,54], which collectively degrade the structural and spatial attributes that define suitable habitat. Consequently, effective conservation of *T. temminckii* requires management approaches that explicitly prioritize the preservation, restoration, and connectivity of fine scale habitat mosaics. The insights generated here, by elucidating how specific microhabitat features support different life stages, provide an essential theoretical and empirical foundation for the development of targeted, evidence based conservation strategies aimed at sustaining the species long term population viability.

Based on our findings regarding the microhabitat selection patterns of *T. temminckii*, we propose several targeted conservation management recommendations for Laojunshan National Nature Reserve and comparable subtropical montane forests. First, management priority should be placed on preserving bamboo patches and intact forest floor litter layers on gentle to moderate slopes, as these microhabitat attributes are strongly associated with nesting success and chick survival. Second, maintaining or restoring the vertical structural complexity of forests, particularly a multi layered canopy with a well-developed understory, is essential for providing seasonal shelter, foraging substrates, and refuge from predators during the breeding period.

Although these management insights are derived from a localized population with a single breeding season, they underscore the importance of seasonally informed and habitat specific conservation strategies. We acknowledge that these findings require validation across the species’ wider distribution and over multiple years to assess their broader applicability and temporal consistency. Furthermore, we also acknowledge that our conventional plot based habitat sampling approach may not fully capture proportional habitat use, subtle selection behaviors, or potential variation among demographic groups such as males versus females or adults versus juveniles. Future research incorporating advanced animal tracking technologies, including high resolution GPS telemetry or accelerometry, could provide deeper insights into fine scale habitat selection processes, movement ecology, and behavioral plasticity across seasons and demographic classes. Such data would substantially enhance conservation planning not only for *T. temminckii* but also for other understory dependent avian species inhabiting similarly fragmented forest ecosystems.

## 5. Conclusions

This study elucidates the key environmental determinants governing breeding microhabitat selection in the Temminck’s Tragopan. Our findings demonstrate that the species exhibits a strong preference for a distinct suite of habitat characteristics, including moderate slopes, predominantly shady aspects, close proximity to perennial streams, a well-developed bamboo understory, and a sparse herbaceous layer. Collectively, these structural and topographic features create a microhabitat template that fulfills critical reproductive requirements by enhancing concealment from predators, ensuring reliable access to food resources, and providing stable and sheltered sites for nest establishment and incubation. Consequently, effective conservation of this vulnerable pheasant necessitates the targeted preservation and management of forest stands that maintain this specific and integrated combination of habitat attributes, as they are fundamental to supporting successful reproduction and long term population persistence.

## Figures and Tables

**Table 1:** Comparisons of microhabitat variables of Temminck’s tragopan (*Tragopan temminckii*) between used microhabitats and control quadrats during the breeding season.

Variable	Microhabitat (n = 40)	Control Quadrat (n = 40)	*p*
Elevation (m)	1552.3 ± 18.84	1541.58 ± 17.81	0.661
Slope (°)	29.75 ± 1.34	21.18 ± 2.53	<0.001 ***
Aspect	/	/	0.665
Tree height (m)	12.50 ± 0.83	5.08 ± 1.14	<0.001 ***
Tree coverage (%)	47.85 ± 3.45	16.62 ± 4.25	<0.001 ***
Shrub height (m)	4.52 ± 0.17	3.62 ± 0.26	0.004 **
Shrub coverage (%)	43.60 ± 2.50	44.95 ± 4.53	0.740
Litter coverage (%)	76.22 ± 1.85	61.92 ± 2.94	<0.001 ***
Herb height (cm)	23.38 ± 1.38	28.80 ± 2.16	0.038 *
Herb coverage (%)	21.90 ± 2.53	42.12 ± 3.92	<0.001 ***
Bamboo height (m)	3.73 ± 0.33	0.95 ± 0.27	<0.001 ***
Bamboo coverage (%)	48.69 ± 6.09	15.58 ± 4.38	<0.001 ***
Distance to stream (m)	26.05 ± 4.24	47.88 ± 7.15	0.011 *
Distance to road (m)	727.53 ± 136.00	729.62 ± 136.69	0.992
Distance to forest (m)	25.60 ± 9.34	27.62 ± 7.22	0.370

Note: * *p* < 0.05; ** *p* < 0.01; *** *p* < 0.001.

**Table 2:** Principal component loading matrix of microhabitat variable of Temminck’s tragopan (*Tragopan temminckii*) during the breeding season.

Microhabitat Factors	RC1	RC2	RC3	RC4	RC5	RC6
Elevation	−0.083	−0.150	0.029	0.647	−0.361	0.306
Slope	0.069	0.181	−0.133	0.045	0.083	0.889
Aspect_sin	−0.407	0.271	0.002	0.330	0.086	−0.289
Aspect_cos	−0.082	0.284	0.184	0.523	−0.059	−0.084
Tree height	0.170	0.874	0.106	−0.019	−0.102	0.148
Tree coverage	0.188	0.844	0.128	0.029	−0.163	0.112
Shrub height	−0.005	0.651	−0.165	0.211	0.404	−0.143
Shrub coverage	0.007	0.136	−0.119	0.135	0.820	−0.098
Litter coverage	0.325	0.135	−0.192	0.196	−0.657	−0.244
Herb height	−0.190	−0.144	−0.139	0.117	0.757	0.057
Herb coverage	−0.425	−0.055	0.004	−0.219	0.636	0.017
Bamboo height	0.892	0.267	−0.032	−0.054	−0.212	0.028
Bamboo coverage	0.909	0.132	−0.030	−0.026	−0.167	0.023
Distance to stream	0.024	−0.024	0.895	0.147	−0.043	−0.110
Distance to road	0.062	−0.010	0.089	0.714	0.378	−0.035
Distance to forest	−0.077	0.144	0.860	0.068	−0.071	−0.015
Eigenvalue	3.636	2.411	2.022	1.279	1.074	1.063
Contribution (%)	22.72	15.07	12.64	7.99	6.71	6.65
Cumulative contribution (%)	22.72	37.79	50.43	58.42	65.14	71.78

**Table 3:** The parameters of logistic regression models of Temminck’s tragopan microhabitat use during the breeding. Only model with ΔAICc ≤ 2 were included.

Principal Component	Parameter Estimates	Standard Error	Lower 95%CI	Upper 95% CI	*p*
Intercept	−0.114	0.540	−1.194	0.965	0.835
PC1	4.023	1.209	1.603	6.442	0.001 **
PC2	3.321	1.038	1.245	5.398	0.002 **
PC3	−2.039	1.123	−4.286	0.208	0.075
PC4	0.333	0.524	−0.473	1.850	0.534
PC6	0.688	0.581	−0.716	1.381	0.246

Note: ** *p* < 0.01.

## Data Availability

The data presented in this study are available on request from the corresponding author.
